# Liquid-liquid separation in gut immunity

**DOI:** 10.3389/fimmu.2024.1505123

**Published:** 2024-12-10

**Authors:** Zhaoyang Wang, Lili Zhou, Xiaolan Zhong, Yiguo Jiang, Zhentao Zhang, Wanglin Li

**Affiliations:** ^1^ Department of Gastrointestinal Surgery, Huadu District People’s Hospital, Guangzhou, China; ^2^ Biology, School of Public Health, Guangzhou Medical University, Guangzhou, China; ^3^ Department of Cell Biology, Jinan University, Guangzhou, China; ^4^ Department of Gastroenterology, Huadu District People’s Hospital, Guangzhou, China; ^5^ State Key Laboratory of Respiratory Disease, The First Affiliated Hospital of Guangzhou Medical University, Xinzao, Guangzhou, China; ^6^ Obstetrics and Gynecology Department, Binzhou Medical University Hospital, Binzhou, Shandong, China; ^7^ Guangzhou Key Laboratory of Digestive Diseases, Guangzhou Digestive Disease Center, Guangzhou First People’s Hospital, School of Medicine, South China University of Technology, Guangzhou, Guangdong, China

**Keywords:** liquid-liquid separation, gut immunity, condensate, immune cell, inflammatory bowel disease

## Abstract

Gut immunity is essential for maintaining intestinal health. Recent studies have identified that intracellular liquid-liquid phase separation (LLPS) may play a significant role in regulating gut immunity, however, the underlying mechanisms remain unclear. LLPS refers to droplet condensates formed through intracellular molecular interactions, which are crucial for the formation of membraneless organelles and biomolecules. LLPS can contribute to the formation of tight junctions between intestinal epithelial cells and influence the colonization of probiotics in the intestine, thereby protecting the intestinal immune system by maintaining the integrity of the intestinal barrier and the stability of the microbiota. Additionally, LLPS can affect the microclusters on the plasma membrane of T cells, resulting in increased density and reduced mobility, which in turn influences T cell functionality. The occurrence of intracellular LLPS is intricately associated with the initiation and progression of gut immunity. This review introduces the mechanism of LLPS in gut immunity and analyzes future research directions and potential applications of this phenomenon.

## 1 Introduction

The gut is one of the parts of the human body that has the most frequent contact with the external environment, facing daily threats from a multitude of bacteria, viruses, fungi, and other pathogens (1, 2). The immune system plays a crucial role in recognizing and eliminating these pathogens, thereby ensuring intestinal health (2, 3). It maintains the integrity of the intestinal mucosa and prevents the invasion of harmful substances by regulating the tight junctions (TJs) between intestinal epithelial cells (4). Additionally, the immune system participates in the metabolic processes of nutrients and regulates their utilization and storage (5, 6). Gut immunity is closely associated with a variety of diseases (7, 8). A thorough investigation into the mechanisms underlying intestinal immunity holds significant importance for the prevention, diagnosis, and treatment of these conditions. An increasing number of studies have demonstrated that liquid-liquid phase separation (LLPS), which has garnered significant attention recently, also plays a role in the intestinal immune response. LLPS refers to two different phases formed by the spontaneous aggregation of biological macromolecules (proteins, nucleic acids, etc.) in eukaryotic cells. It is an important mechanism that enables specific biomolecules to accumulate in local areas within cells and form membraneless structures with unique physical and chemical properties and biological functions. LLPS is involved in various biological processes (9, 10). However, the mechanism of LLPS involvement in gut immunity is still unclear. This review will focus on gut immunity and LLPS, providing a comprehensive analysis of the specific mechanisms through which LLPS regulates gut immune responses.

## 2 Molecular mechanism of liquid-liquid phase separation in gut

### 2.1 Formation of liquid-liquid phase separation in gut

**Figure 1:**
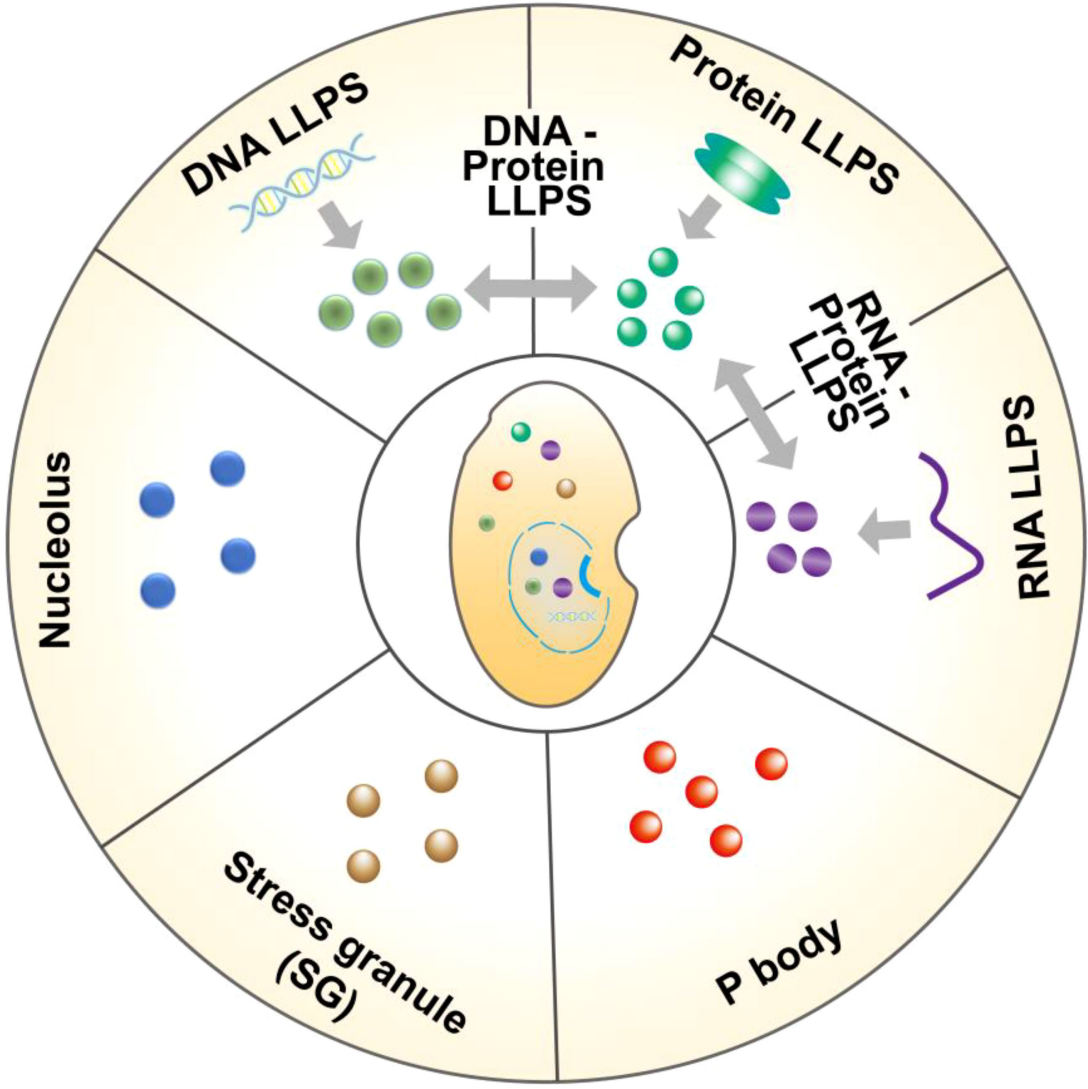
Different forms of liquid-liquid separation in gut cells. LLPS in gut cells are formed by the interaction of macromolecular proteins, RNA, DNA, etc., including protein and protein, DNA and protein, DNA and DNA, RNA and RNA, RNA and protein. In addition, LLPS also participates in the formation of some membraneless organelles in cells, such as P body, stress granule (SG), nucleolus, etc.

LLPS is a fascinating phenomenon that typically arises from the interactions among various molecules within cells, including proteins, RNA, and DNA. These distinct molecules aggregate through multivalent interactions, van der Waals forces, hydrogen bonds, and other interactions, leading to the formation of different phase states (11). LLPS also exists in different cells in the gut. In intestinal epithelial cells, a variety of proteins participate in LLPS, among which zonula occludens 1 (ZO-1) protein and other barrier related proteins can produce LLPS, further forming the physical barrier of the gut to prevent pathogens and harmful substances from entering the body. ZO-1’s ability to generate LLPS is attributed to weak polyvalent interactions between the disordered regions (IDRs) it possesses (12). These multivalent interactions regulate the LLPS dynamics of protein molecules and determine which components are segregated into condensates (**Figure 1**). Stable multivalent interactions required for LLPS can be mediated by interactions among multiple folded domains or short linear motifs, such as the Src homology domain 3 (SH3) module or proline-rich motifs (PRMs) (13). Furthermore, an increasing number of studies have demonstrated that RNA also plays a significant role in regulating the occurrence of LLPS in gut cells (**Figure 1**). RNA can serve as a molecular scaffold to bind multiple RNA-binding proteins (RBPs), facilitating the formation of a dynamic network of RBPs, which results in phase-separated droplets (14, 15). Furthermore, LLPS can also occur between DNA-DNA and DNA-protein (**Figure 1**). For example, when gut cells are damaged, the DNA damage response (DDR), repair factors 53BP1 (p53-binding protein 1) and RAD52 (functional analog of human BRCA2) can form LLPS at the site of DNA damage and quickly complete DNA damage repair. It has been found that the 53BP1 repair chamber at the DNA damage site shows the characteristics of LLPS, and the destruction of 53BP1 condensate reduces the activation of p53 target genes (such as p21). Once the phase separation of 53BP1 was impaired, both p53 stabilization and p21 expression induced by DNA damage were significantly reduced (16). In addition, Rad52 proteins at different DNA damage sites can aggregate into droplets and fuse into repair center droplets through the action of pti-DIMs induced by small DNA damage. The resulting droplet binds the repair center to a longer period of DIM-mediated mobilization of damaged DNA for repair (17). It can be seen that droplets formed through LLPS can concentrate repair factors and DNA, providing an efficient microenvironment for DNA repair (18).

Recent evidence suggests that LLPS is a key mechanism for the formation of membraneless organelles in gut cells. For example, P bodies are membraneless structures in the cytoplasm that form through LLPS and play an important role in RNA metabolism and transport (19) (**Figure 1**). Similarly, the nucleolus, another structure formed by LLPS, is capable of accumulating substantial amounts of rRNA and proteins, thereby playing a pivotal role in ribosome biosynthesis (20) (**Figure 1**). Furthermore, when cells experience stress (such as oxidative stress or heat shock), LLPS helps cells respond quickly to form stress granules (SG) that temporarily store unfolded proteins and other cellular components, thereby protecting cells from damage (11, 21) (**Figure 1**). LLPS enables cells to create closed chambers that isolate their contents from the external environment, allowing biological reactions to occur quickly and efficiently. As research progresses, our understanding of LLPS will deepen, revealing the complexity of life processes and opening up new avenues for the treatment of gut-related diseases.

### 2.2 Factors affecting liquid-liquid separation

Numerous factors influence LLPS, including light, temperature, pH, salt concentration, and post-translational modifications (PTMs) (22). The most common method for inducing LLPS is by altering the concentration of salt ions. Certain proteins, such as DEAD-box helicase 3 X-linked (DDX3X), exhibit a propensity for LLPS at elevated salt concentrations, as salt ions can modify the weak interactions between molecules, thereby influencing the occurrence of phase separation. Furthermore, ATP plays a significant role in the assembly of aggregates and serves as a water-soluble growth aid, mitigating protein aggregation associated with LLPS (23). Additionally, some findings indicate that various ATP-driven remodeling complexes regulate SG (24). First, ATP is recognized as a key driver of phase separation. Moreover, ATP helps maintain protein concentration within the particle, and when protein levels are high, ATP may counteract the tendency of intrinsically disordered regions (IDRs) to form amyloid fibers. Importantly, PTM plays a significant role in regulating protein LLPS. The currently identified PTMs that influence LLPS include phosphorylation, methylation, acetylation, and ubiquitination (25–27). These modifications promote or inhibit LLPS by altering the charge distribution and hydrophobic characteristics of the disordered regions within the protein. Increased levels of tau acetylation do not facilitate the formation of LLPS, however, elevated levels of tau phosphorylation enhance LLPS (28, 29). Additionally, the increased phosphorylation of ZO-1, a crucial protein in the intestinal barrier, also triggers LLPS, thereby further promoting the formation of TJs (30). An increase in temperature will enhance the thermal motion of molecules, thereby influencing the interaction forces between them. Additionally, temperature plays a crucial role in regulating LLPS (31, 32). *In vitro* studies have demonstrated that initially condensed protamine and protamine-polyvalent ionic complexes dissociate with rising temperatures, however, their aggregation resumes upon cooling (33). This observation suggests that LLPS is contingent upon specific temperature conditions.

Understanding how these factors influence LLPS is essential for gaining deeper insights into biochemical reactions and physiological functions within gut cells. Additionally, this knowledge may offer valuable clues for investigating the mechanisms underlying related diseases.

## 3 Liquid-liquid separation is involved in the process of gut immunity

### 3.1 Liquid-liquid phase separation in gut immunity

**Figure 2:**
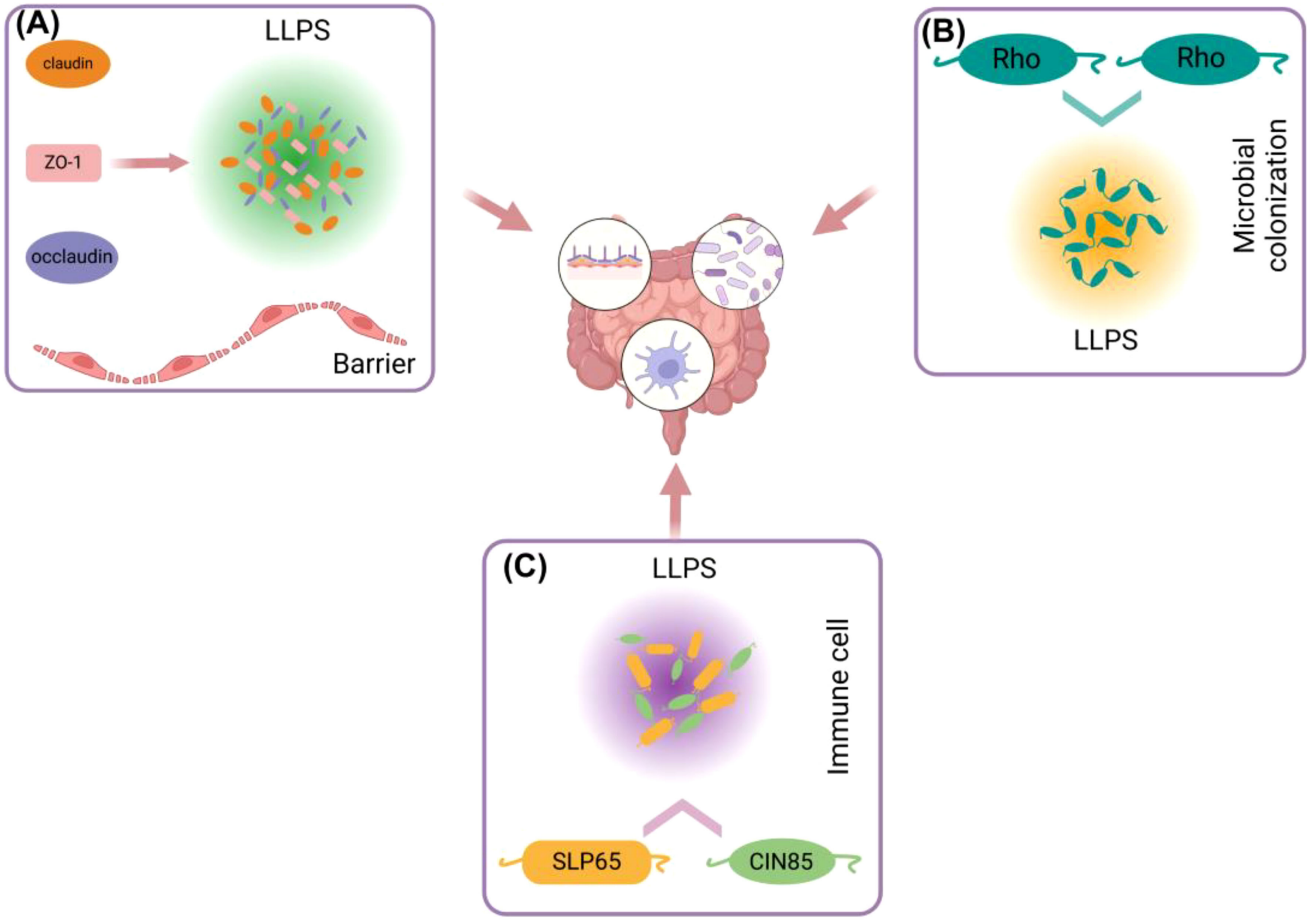
Different ways in which liquid-liquid separation is involved in gut immunity. **(A)** ZO-1, Claudin, Occludin and other proteins in intestinal epithelial cells can use ZO-1 as a scaffold to generate LLPS and participate in the formation of intestinal barrier. **(B)** LLPS between Rho proteins in gut microorganisms is involved in colonization of gut microorganisms. **(C)** LLPS of SLP65 and SIN85 proteins in gut immune cells (B cells) is involved in gut immune regulation.

The gut immune system primarily comprises intestinal epithelial cells, intestinal lymphocytes, Paneth cells, and intestinal microbiota, which collectively protect the host from pathogens, maintain immune tolerance, and promote tissue repair (34). As a significant regulatory mechanism, LLPS may play a crucial role in the signaling of gut immunity, the regulation of immune responses, and the interaction with microbiota. Within the barrier formed by intestinal epithelial cells, the occurrence of ZO-1 LLPS, a critical protein in TJs, may regulate barrier function. Specifically, the disordered domain at the C-terminus promotes ZO-1 LLPS and facilitates the recruitment of claudin-1 and occludin proteins into the condensate (30), thereby enhancing the formation of TJs and maintaining the integrity of the intestinal barrier (**Figure 2A**). Furthermore, it has been observed that bacteria utilize LLPS to improve their adaptability to the mammalian gut. For instance, the adaptability of the probiotic Bacteroides polymorphoides, which exists in symbiosis with humans, in the mouse gut is dependent on the LLPS of the transcription termination factor Rho (35). This finding suggests that LLPS is essential for the immune system established in the gut by intestinal microbes (**Figure 2B**).

In the gut, immune cells, including dendritic cells and T cells, form specific intracellular structures through LLPS, such as SG and nuclear bodies (36). Research indicates that LLPS plays a crucial role in T cell receptor (TCR) signaling and DNA recognition within immune cell signaling in the gut. In this context, LLPS functions as a reaction vessel, facilitating the mobility of persistent substrates and products, which in turn enhances signal transduction and enzymatic reactions (37). For instance, the formation of T cell microclusters, first described in the late 1990s, depends on ligand binding and phosphorylation, involving transmembrane receptors such as TCR, CD28, and PD1, kinases LCK and ZAP70, adapter proteins LAT and GRB2, as well as GADS (also known as GRAP2), SLP76 (also known as LCP2), and NCK1, and enzymes SOS1, PLCγ1, and CBL (38, 39). The composition of these clusters is heterogeneous and dynamic, with cluster components typically exhibiting higher densities and lower mobilities compared to their surrounding environment. High density can enhance the likelihood of molecular interactions within the cluster, while low mobility can facilitate the minimum binding time required for such interactions. Conversely, the scaffolding protein SLP65, its binding partner CIN85, and liposomes (spherical vesicles) can form liquid-like condensates in the cytosol of resting B cells, thereby regulating signaling (**Figure 2C**). The helical domain trimerizes, further promoting the formation of LLPS. Upon B cell stimulation, the condensate migrates towards the cell membrane and further phosphorylates SLP65, triggering downstream signaling pathways, including RAS activation, NF-κB, and calcium influx, among others (40). Although no LLPS information directly related to Paneth cells has been found, antimicrobial peptides secreted by Paneth cells are likely to participate in the formation of LLPS. Studies have shown that antimicrobial peptides (such as LL-III) can regulate LLPS formed by P granuloprotein LAF-1 and RNA (41). On the other hand, LLPS is also able to regulate the overactivation of immune cells, a function highlighted in DNA sensor clocks, e.g., [cyclic GMP-AMP] synthase - stimulator of interferon (IFN) genes - IFN regulatory factor 3 and IFI16 (IFNγ inducible protein 16) pathways (42, 43). LLPS can isolate substances in the condensate, increase the concentration of proteins, for example, prompt STING to activate a negative feedback mechanism at high levels of 3’,5’-cyclic adenosine monophosphate (cAMP), thereby stopping the overactivation of immunity (44).

**Figure 3:**
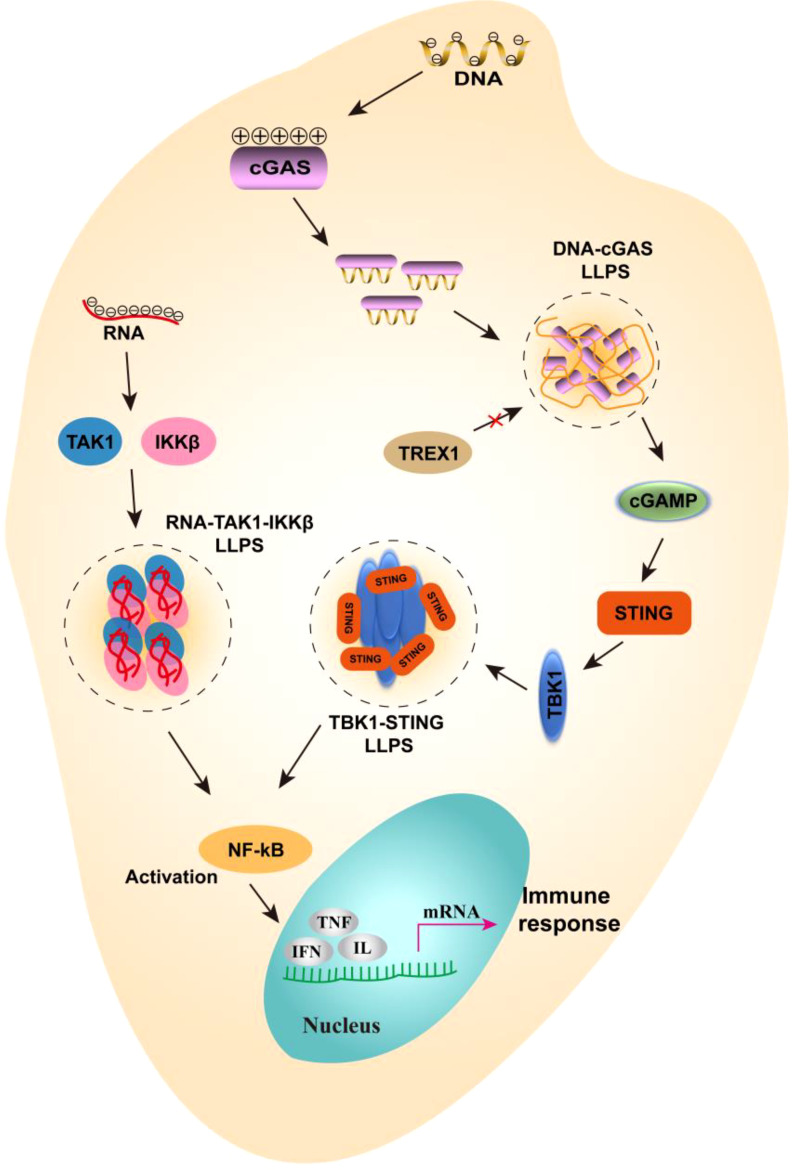
The mechanism of liquid-liquid separation involved in NF-κB signaling pathways. In gut immune cells, negatively charged DNA can bind to positively charged cGAS to form LLPS, which prevents the degradation of DNA by TREX1 and passes the signal downstream to cGAMP. Further, cGAMP activated STING and TBK1, which interact to form LLPS, thereby promoting the activation of NF-κB. On the other hand, when RNA viruses infect the gut, viral RNA can bind to TAK1 and IKKβ to form LLPS, further promoting the activation of NF-κB. Eventually, inflammatory factors are expressed and an immune response is generated.

In conclusion, LLPS serves as a highly efficient regulatory mechanism of the gut immune response. However, dysregulation of LLPS can also result in inflammation and disease. Therefore, a comprehensive study of the role of LLPS in intestinal immunity will provide a significant theoretical foundation and practical guidance for understanding the immune mechanisms and developing new therapies.

### 3.2 Liquid-liquid phase separation is involved in immune signaling pathways

In addition to its roles in intestinal barrier formation, gut microbial colonization, and the clustering of gut immune cell membranes, LLPS also plays a critical role in regulating immune signals in intestinal cells. For instance, LLPS can modulate the NF-κB and cyclic GMP-AMP synthase (cGAS)-stimulator of interferon genes (STING) pathways in these cells (45). The RelA subunit of NF-κB is thought to contain an intrinsic disordered region that facilitates its participation in LLPS, thereby promoting the assembly of signaling complexes and enhancing the efficiency of signal transduction (46). Furthermore, in the context of intracellular DNA sensing, cGAS is adept at detecting abnormal double-stranded DNA (dsDNA) that may arise from pathogens or from damage to nuclear and mitochondrial DNA. This interaction results in the formation of liquid condensates that enhance cGAS activity by shielding the DNA from degradation by the exonuclease TREX1 (42). Additionally, cGAS catalyzes the synthesis of 2’, 3’-cyclic GMP-AMP (cGAMP), which activates STING, initiates downstream signaling pathways, and induces the expression of type I interferon and anti-inflammatory cytokines (47, 48) (**Figure 3**). During intestinal immunity, certain viruses utilize LLPS to modulate immune responses by isolating signaling molecules. For instance, the thrombocytopenia syndrome virus (SFTSV) encodes non-structural (NS) proteins that form membraneless liquid condensates, referred to as inclusion bodies, which facilitate viral genome replication and alter antiviral immune responses to evade host immune surveillance (49). Recent studies have shown that following infection with respiratory syncytial virus (RSV), the NF-κB subunit p65 is rapidly sequestered into perinuclear cytoplasmic spots, thereby preventing its translocation to the nucleus and subsequent activation of pro-inflammatory cytokine genes, as well as the downstream transcription of other antiviral genes (50). Conversely, LLPS can also promote the activation of NF-κB in the context of viral infection. Recent findings indicate that the N protein of SARS-CoV-2 interacts with viral RNA to undergo LLPS, leading to the formation of a functional membraneless organelle that recruits TAK1 and IKKβ complexes, thereby enhancing NF-κB activation (49) (**Figure 3**). These examples underscore the emerging concept that LLPS may play both positive and negative roles in the regulation of signaling pathways. It is evident that LLPS does not simply promote or inhibit the intestinal immunity, and it serves as a complex regulatory mechanism that maintains a delicate balance.

## 4 Research progress of liquid-liquid phase separation in gut inflammatory diseases

Inflammatory bowel disease (IBD) is a chronic immune-mediated condition that affects the gastrointestinal tract (51). This disease is believed to arise from interactions among environmental, microbial, and immune-mediated factors in genetically susceptible individuals (52). The intestinal flora plays a crucial role in the onset and progression of IBD (53). It is known that bacteria require LLPS to adapt effectively to the mammalian intestine. In this context, Eduardo A, Groisman et al., determined that the adaptability of Bacteroides thetaiotaomicron in the mouse gut is facilitated by a unique domain of its transcription terminator Rho, which is essential for LLPS (35). Concurrently, some researchers have found that fecal transplantation of B. thetaiotaomicron in a dextran sulfate sodium (DSS)-induced IBD model can significantly alleviate IBD symptoms (54). G protein-coupled receptors (GPCRs) are critical components of the immune system, and emerging evidence suggests a significant association between GPCRs and the pathogenesis of IBD (55). The study revealed that the IBD-associated variant of G protein-coupled receptor 65 impairs signaling and disrupts key functions involved in inflammation (56). Signal transduction molecules such as GPCRs, G proteins and downstream effectors can form signal transduction complexes through LLPS. This complex is highly dynamic and adaptable, allowing for rapid responses to changes in signals both inside and outside the cell. Furthermore, local compartments formed by LLPS can elucidate how multiple G protein-coupled receptors achieve specificity despite their reliance on the common messenger cAMP (57). The LLPS of ZO-1 protein is a key factor driving the formation of tight junction, and the integrity of intestinal barrier is the basis for maintaining immune function (30, 58). Therefore, the LLPS of protein is closely related to the occurrence and development of IBD, and the LLPS of regulatory protein has become a possibility to treat IBD.

The complexity and dynamics of the intestinal microenvironment play a crucial role in host health. Factors such as microbial communities, intestinal epithelial cells, the immune system, and the extracellular matrix, along with pH value, collectively influence intestinal homeostasis and the onset of diseases (59, 60). Variations in pH not only affect the charge state of molecules but also modify interactions, including electrostatic interactions, hydrophobic interactions, and hydrogen bonds between them. As pH increases or decreases, the attractive or repulsive forces between molecules change, thereby impacting their aggregation behavior. For instance, at certain pH levels, molecules may remain dispersed due to electrostatic repulsion, while under different pH conditions, mutual attraction may lead to LLPS (61). In the gut, the normal pH range typically falls between 6 and 7.5, however, during inflammation or disease, pH can deviate significantly from this range. Such changes may induce LLPS of specific proteins in the gut, resulting in aggregate formation that adversely affects gut function and immune response (20, 62). Moreover, oxidative stress can promote LLPS by altering intracellular environmental conditions, such as pH, temperature, and ion concentration. For example, oxidative stress may lead to PTMs of certain proteins, such as phosphorylation or ubiquitination, which can influence the proteins’ capacity to interact and LLPS (63). Intestinal inflammation is frequently associated with heightened oxidative stress (64). LLPS can lead to the formation of SG within cells, which aggregate stress-related proteins and assist cells in managing oxidative damage. In this context, LLPS functions as a dynamic regulatory mechanism that can swiftly respond to environmental changes, thereby safeguarding cells from harm. It is evident that LLPS is a significant biological phenomenon that plays a complex role in intestinal inflammation. Furthermore, LLPS may be crucial in inflammatory responses by regulating immune signaling pathways, responding to oxidative stress, and influencing the cellular microenvironment.

## 5 Liquid-liquid phase separation as a potential target for the treatment of gut diseases

With a deeper understanding of the LLPS mechanism, researchers have begun to explore its potential applications in medicine and biotechnology. In particular, LLPS modulators may emerge as a new class of therapeutic drugs aimed at modulating intestinal immunity and reducing inflammation. LLPS facilitates the rapid aggregation of signaling molecules and effector proteins by immune cells upon encountering pathogens, thereby enhancing the efficiency and speed of immune responses. Wang and Zhou investigated how LLPS influences the body’s response to infection by regulating innate immunity, suggesting that this mechanism may provide a foundation for the development of novel immunomodulatory treatments (65). LLPS modulators are compounds that affect LLPS behavior within cells. These regulators can alter the function of immune cells by modifying the physical and chemical properties of the intracellular environment, thereby influencing protein aggregation and LLPS. Studies focusing on intestinal immunity have demonstrated the potential of LLPS modulators in this context. The gut represents a rich immune environment, populated by numerous immune cells and microbial communities (66). LLPS modulators may alleviate intestinal inflammation by enhancing the barrier function of intestinal epithelial cells and improving the responsiveness of immune cells. Some research has indicated that LLPS in the gut could play a significant role in the pathogenesis of IBD (35, 67). By modulating LLPS, researchers aim to improve intestinal inflammatory responses, potentially providing new treatment options for patients with IBD. Future studies should further investigate the specific mechanisms of action of LLPS modulators in immune cells, including their effects on signaling pathways and gene expression. This will provide a theoretical basis for the design of optimized regulators.

LLPS is closely linked to the occurrence and progression of various diseases, including neurodegenerative disorders and cancer (68, 69). In these conditions, the abnormal LLPS of proteins can lead to the formation of detrimental aggregates that disrupt normal cellular functions. As researchers deepen their understanding of LLPS, they are investigating its potential applications in drug development. In the past, it was widely believed that small molecule drugs were evenly distributed after entering cells. However, studies on LLPS have shown that this phenomenon significantly affects the distribution of small molecule drugs (70, 71). Consequently, it is essential not only to deliver these drugs to the appropriate cells but also to ensure their localization within the correct organelles. Furthermore, targeted LLPS has emerged as a promising avenue for drug development. On one hand, small molecular compounds that interact with proteins in membraneless organelles may either promote or inhibit LLPS, and are currently being screened for this purpose. On the other hand, research has demonstrated that various enzymes, including ATPases, helicases, and ubiquitin ligases, which regulate protein post-transcriptional modifications, have the potential to influence LLPS (13, 72). Therefore, inhibitors or agonists of these enzymes—particularly those targeting protein post-transcriptional modification—represent small molecules with significant potential for modulating LLPS. However, many questions remain unanswered: Should we promote or inhibit LLPS in diseases such as cancer or IBD? Can specific small molecule drugs effectively enter and exit membraneless organelles? Furthermore, can these drugs precisely and specifically regulate the occurrence of LLPS? It can be asserted that research on phase separation presents both opportunities and challenges.

## 6 Conclusions and future prospects

LLPS facilitates the concentration and regulation of immune factors by intestinal immune cells, including macrophages and lymphocytes, through the formation of aggregates (73). This process enables these cells to rapidly produce and release cytokines, thereby effectively responding to pathogens and inflammation. In the gut, immune cells can generate specific immune condensates via phase separation mechanisms, which are capable of accommodating and processing various immunogens, including viruses and antigens. This mechanism is particularly crucial for the activation of innate immunity and enhances the rapid activation of effector cells, such as phagocytes. Macrophages play a pivotal role in maintaining intestinal immune homeostasis, with LLPS modulating their functionality to balance pro-inflammatory and anti-inflammatory responses (74). For instance, certain macrophage subsets may promote inflammation through LLPS mechanisms in inflammatory environments, while others can suppress inflammation and restore intestinal homeostasis. Furthermore, LLPS not only influences the aggregation of immune factors but also impacts the activity of intracellular signal transduction pathways. By regulating the distribution and aggregation of signaling proteins, cells can transmit information more efficiently, optimizing the timing and intensity of immune responses. Although there is a preliminary understanding of the role of LLPS in cell signaling, its molecular mechanisms require further exploration. Utilizing high-resolution microscopy and molecular biology tools, researchers can gain deeper insights into the dynamic processes of LLPS and its regulatory mechanisms. Additionally, LLPS may interact with other biological processes within cells, such as the cell cycle and metabolism. Future studies could investigate these interactions to reveal the full impact of LLPS in cell biology. Clinically, intervention targeting phase separation may provide new therapeutic strategies for diseases such as intestinal inflammation. Future studies should focus on the screening of LLPS regulators and their application in animal models and clinical trials.

Although some progress has been made in the study of LLPS in IBD, there are still many unanswered questions. It is unclear whether and how microbial metabolites affect the occurrence of LLPS in IBD. For example, can short chain fatty acids (SCFAs), which are important metabolites produced by intestinal microorganisms in the fermentation of dietary fiber, influence intestinal barrier function and inflammatory response by regulating the activity or interaction of LLPS-related molecules? Studying the effects of microbial metabolites on LLPS will help reveal new mechanisms of action of gut microbes in the pathogenesis of IBD. LLPS is not only associated with IBD but also plays a significant role in the onset and progression of various diseases, including Alzheimer’s disease, Parkinson’s disease and tumor diseases (29, 75, 76). In the brains of individuals with Alzheimer’s disease, there is an abnormal accumulation of amyloid beta (Aβ) and tau proteins, Research has demonstrated that these proteins can form membraneless organelle-like condensates through LLPS. These aggregates may further promote protein misfolding and aggregation, exacerbate neurotoxicity, and lead to neuronal damage and cognitive dysfunction (77). Additionally, LLPS is crucial in tumor cells, where certain oncogenic and tumor suppressor proteins can form condensates via this process, thereby regulating gene expression and signal transduction, and influencing biological behaviors such as proliferation, apoptosis, invasion, and metastasis of tumor cells. For instance, transcription factors like c-Myc and p53 can create condensates through LLPS, modulating the expression of downstream genes and contributing to tumor initiation and progression (78, 79). Given the significant role of LLPS in tumor cells, researchers may consider phase separation-related biomarkers as valuable tools for tumor diagnosis and prognosis. These markers can aid clinicians in better understanding the biological characteristics of tumors and in developing personalized treatment strategies. Furthermore, due to the critical importance of LLPS in immune cell function, investigating its role within the tumor microenvironment could yield new insights for optimizing immunotherapy. By modulating the LLPS behavior of immune cells in this context, it may be possible to enhance their ability to target tumors, thereby improving the efficacy of immunotherapeutic approaches. Overall, biomarker discovery based on LLPS provides new perspectives and methods for understanding and precisely treating immune-related diseases. With the deepening of research, this field is expected to bring revolutionary changes to clinical medicine.
